# Traditional Korean Medicine-Based Forest Therapy Programs Providing Electrophysiological Benefits for Elderly Individuals

**DOI:** 10.3390/ijerph16224325

**Published:** 2019-11-06

**Authors:** Jiyune Yi, Boncho Ku, Seul Gee Kim, Taegyu Khil, Youngsuwn Lim, Minja Shin, Sookja Jeon, Jingun Kim, Byunghoon Kang, Jongyeon Shin, Kahye Kim, Ah Young Jeong, Jeong Hwan Park, Jungmi Choi, Wonseok Cha, Changseob Shin, Wonsop Shin, Jaeuk U. Kim

**Affiliations:** 1 Department of Forest Therapy, Graduate School of Chungbuk National University, Chungju, Chungbuk 28644, Korea; jiyuneyi@gmail.com (J.Y.); byunghoon21@naver.com (B.K.);; 2 Korea Institute of Oriental Medicine, Daejeon 34054, Korea; 3 Human Anti-Aging Standards Research Institute, Uiryeong, Gyungnam 52151, Korea

**Keywords:** forest therapy, cognitive impairment, dementia, breathing program, walking program, Sasang constitutional medicine, electrophysiology, EEG, HRV, bioimpedance

## Abstract

We aimed to develop forest therapy programs (FTPs) to prevent dementia and related health problems in the elderly population, with the assumption that health benefits are FTP-type specific and depend on the participant’s psychophysiological traits. For this purpose, we developed two distinct FTPs, namely, a guided-breathing meditation program (BP) and a walking program (WP); we adopted the approach of Sasang constitutional (SC) medicine, which categorizes individuals into one of three SC types (SC1, SC2, or SC3) for medical care. The FTPs ran 11 sessions over 11 weeks. We recruited 29/31/28 participants who were 65 years of age or older for the BP/WP/control groups, respectively; obtained electrophysiological measurements via electroencephalogram (EEG), heart rate variability (HRV), and bioimpedance; and analyzed the intervention effects with analysis of covariance. Compared with the control, the BP and WP resulted in benefits for neural activity and parasympathetic nervous activity (PNA), respectively, and both FTPs yielded distinct beneficial effects on bioimpedance. Constitution-specific effects were also present. The SC1- and SC2-type participants gained positive effects in neural activity from the BP and WP, respectively. The SC3-type participants showed improvements in PNA from the WP. In conclusion, for older individuals, both programs conferred health benefits that would help prevent dementia, and the benefits were program-specific and constitution-specific.

## 1. Introduction

The aging of society is a global issue, and South Korea became a member of the ‘aged societies’ as the proportion of its total population accounted for by the elderly population over 65 years reached 14.2% in the ‘2017 Population and Housing Census’ [1]. Along with the increase in the elderly population, degenerative health problems and the related medical costs have become socioeconomic concerns. In particular, the impact of the growing number of people with dementia challenges national and global public health systems [2]. According to the World Health Organization (WHO), the number of individuals with dementia is expected to triple from 50 million to 152 million by 2050, and the global cost of caring for people with dementia was estimated to be US$ 818 billion, which is approximately 1.1% of the global gross domestic product [3]. This report estimates that the cost could be US$ 2 trillion by 2030. Because there is no cure for dementia, the role played by preventive medicine with diverse interventions such as physical exercise, cognitive interventions, social activities, and activities in nature for the elderly population will increase substantially [4].

Studies show that interacting with nature improves the physical and mental health of people [5,6,7,8,9,10], and forest therapy is gaining more recognition as a nature-based therapy [11,12,13]. The term “forest therapy” has been used interchangeably with more traditionally defined “forest bathing” or “Shinrin-yoku”. The effects of forest bathing on human immune function and physiology are well reported in the literature [12,14,15,16]. According to Li et al. [14], a forest bathing trip “involves a visit to a forest field for the purpose of relaxation and recreation” and Park et al. [16] defined the term as “making contact with nature and taking in the atmosphere of the forest: a process intended to improve an individual’s state of mental and physical relaxation”. In these studies, the activities associated with forest bathing are mainly walking, spending time, or simply remaining in the forest environment. More recently, authors have often distinguished forest therapy from forest bathing as a strengthened form added by guided practices programs that could facilitate participant engagement with various therapeutic activities in the forest for restoration, rehabilitation, and wellness [17,18,19]. 

The conditions on which forest therapy can have beneficial effects include anxiety, depression, mood disorders, stress-related symptoms, hypertension, cancer, immune functions, and cognitive function; the various conditions on which forest therapy or forest bathing have therapeutic effects continue to appear in new studies. Among them, depression, hypertension, physical inactivity, diabetes, and obesity are known risk factors for dementia [20]. Therefore, in aged societies, forest therapy programs (FTPs) can play an important role as a preventive or therapeutic intervention for older adults with normal cognition or mild cognitive impairment. In Korea, forest therapies with older populations are of recent interest, but only a few articles exist [21]; the evidence levels are low because of small numbers of participants, preliminary study designs, or short program hours. For the elderly population, lower mobility and degraded physical and cognitive abilities make the forest less accessible, and recent forest therapies performed with older Korean populations have been mostly based on urban forests. Some authors have reported the benefits of depression and stress relief [22,23]; improved resilience, depression, stress, and quality of life [24]; increases in blood melatonin levels [25]; and improved functional fitness and gait patterns [26]. The studies examining the beneficial effects of FTPs for older people are limited [27,28], and little is known about FTPs as a preventive intervention for dementia.

In this work, we aimed to develop FTPs that can be run in urban forest areas for the elderly population to prevent cognitive decline. We hypothesized that different characteristic activities in the forest may induce different health promotion effects and that these effects may also depend on the participant’s psychological preferences and physiological susceptibilities to activity types. To test both hypotheses, we developed two different characteristic FTPs and analyzed the effects of the FTPs on constitution-specific subgroups by following the approach of Sasang constitutional medicine (SCM), in which participants are categorized into one of three Sasang constitutional (SC) types [29,30]. Second, we measured the risk factors for dementia based on electrophysiological methods such as an electroencephalogram (EEG) for brain function, heart rate variability (HRV) for autonomic nervous function, and bioimpedance for body composition and cellular metabolic status. Electrophysiological assessments provide objective outcomes, and they are noninvasive, portable, cost-effective, and easy to implement in the field of forest therapy. More studies are needed regarding the utility of various electrophysiological analyses as assessment methods for diverse FTPs.

## 2. Materials and Methods

### 2.1. FTPs

We developed two FTPs that were two hours long on average per daily session (10:00~12:00) for a total of 11 sessions with one session per week. In choosing an appropriate program duration and session hours for the prevention of dementia, appropriate references are not sufficient in the field of forest therapy. According to a recent meta-analysis, an exercise training period targeting cognitive function in older adults with mild cognitive impairment (MCI) had a range of 6 weeks to 52 weeks of intervention with one session to three sessions per week and 30 minutes to 90 minutes per session, and the effects of these benefits were not conclusive [31]. A recent random clinical trial performed for elderly individuals with MCI used a 12-week resistance exercise program with two sessions per week and one hour per session; the study reported a positive effect on the EEG along with some physical benefits [32]. In recent papers regarding forest therapy for elderly individuals, a 12-week program with one session per week and two hours per session was developed [22,23,25]. In our pilot study, we assumed that the participants would obtain combined benefits from the guided exercise and healing-relaxation components by activities in the forest, and thus chose the minimal number of intervention hours necessary for the least beneficial effects on cognitive and physiological functions for the elderly. 

To increase the effectiveness of the FTPs, we adopted some therapeutic modalities from traditional Korean medicine (TKM) [33]. The first program is called the “Walking Program (WP)”, and its key design point is active walking in the forest to induce sweating and stimulate the acupuncture point of Yongquan (K11) during walking; this acupuncture point is known to benefit cognitive decline, hypertension, blood circulation, and sleep problems, among other conditions [34,35]. The second program is called the “Breathing Program (BP)”, and its key design point is guided-breathing meditation with the simultaneous stimulation of the cervical spine so that the cervical spine is aligned in its optimal posture, which is known to activate the therapeutic Qi and blood flow through the meridian system in TKM [36]; stimulation of the cervical spine is known to increase cerebral blood flow and reduce neuropathic upper limb pain [37,38], among other health-promotion effects. Both FTPs were designed to promote the cognitive and physical health status of the elderly population, and the BP was especially designed as an appropriate FTP for individuals who have difficulties engaging in active physical exercises in nature. Each program was conducted in urban forests located in Cheongju City, Korea (M-forest and B-forest), which were easily accessible by nearby urban dwellers.

The WP consisted of 30 minutes of preparatory activities, 50 minutes of forest walking, 20 minutes of muscle training with a stretchable band, and 20 minutes of closing activities. The preparation activities consisted of different types of clapping (fist, bud, hand, egg, and fingertip); tapping the whole body and several acupuncture points known for dementia prevention, such as Baihui (GV20), Shenting (GV24), Fengchi (GB20), and Taixi (KI3) [39]; and getting acquainted with nature. Participants taped red beans at Yongquan (K11) [34] to both feet so that the Yongquan could be stimulated by acupressure during walking, and they walked at a tempo that was perceived as slightly intense exercise (13 to 14 points on the Borg rating of perceived exercise in the first three sessions, increasing up to 16~17 points in later sessions [40]). 

The BP consisted of 30 minutes of a preparatory session, 30 minutes of guided-breathing meditation, 20 minutes of slow forest walking, 20 minutes of muscle training with a stretchable band, and 20 minutes of closing activities. The major difference from the WP was the guided-breathing meditation session. It consisted of three guided-breathing parts with different postures and motions, with deep breathing in and out (hypogastric breathing), and induced the stimulation of the cervical spine so that the cervical spine could be aligned towards its optimal posture to activate the therapeutic Qi and blood flow through the meridian system [36] and to increase the cerebral blood flow and cognitive improvement [37].

### 2.2. Sasang Constitutional Medicine

SCM is a branch of TKM and is well established as a standard diagnosis and treatment modality [41]. SCM classifies individuals into one of four SC types, namely, Tae-Eum (TE, Greater Yin), So-Eum (SE, Lesser Yin), So-Yang (SY, Lesser Yang), or Tae-Yang (TY, Greater Yang), and describes the respective physiology, pathology, therapeutic, and health preservation methods [29,30]. According to the theory of SCM, the type-specific clinical symptoms include indigestion, sweat, sleep, urination, and defecation, along with other biopsychological traits. The TY type and TE type are in seesaw relation, and the SY type and SE type are in another seesaw relation with regard to the underlying metabolism. The TY type features strong sympathetic activation, but weak anabolism and energy storage, whereas the TE type features strong anabolism and energy savings, but weak sympathetic activation. On the other hand, the SY type is strong in food intake and digestion, but weak in waste discharge, and the SE type is strong in waste discharge, but weak in food intake and digestion [42]. 

As a recent study reported that the TE-type population accounted for 39.2% of the total 3700 participants, the SE type accounted for 27.1%, the SY type accounted for 33.7%, and the TY type accounted for less than 0.1% in Korea [43]—modern clinical reports on the TY type are very rare. For this reason, the KS-15 questionnaire, which we used to classify the SC types, is a short form with only 15 questions used to classify individuals into the TE, SE, or SY type [44]. As the major features of SCM are described in detail in some classic books and review papers (e.g., the works of [41,45]), we would like to address some weak features of each SC type related to the assumptions involved in composing FTPs. For the TE type, perspiration is a sign of good health, and the absence of perspiration is an unhealthy sign. The SY type is characterized by a hot, unstable, and easily bored temperament and less developed muscles in the lower body parts. On the other hand, the SE type features a negative and nervous mind and less developed muscles in the upper body [29]. We hypothesized that the FTP would be more effective if it can complement or improve the weak psychophysiological features of each SC type, as follows: a low or intermediate intensity of exercise, with upper body exercises, or meditative walk for the SE type; walking/strolling, breathing control, meditation, and lower body part exercises for the SY type; and intermediate to strenuous exercise that induces sweating for the TE type.

### 2.3. Subjects and Study Protocol

The FTPs ran from 8 August 2018 to 14 November 2018. We aimed to recruit 30 participants for each FTP, with a total of 90 elderly participants aged 65 years or older, including 30 participants in the control group (CN) (30 participants in the WP, 30 in the BP, and 30 in the CN). In this study, the participants in the control group received no intervention or treatment related with activities in the forest. Participants were volunteer county dwellers satisfying the following criteria:

- not diagnosed with dementia
- without any restrictions on outdoor activity, including walking for more than three hours
- able to communicate and complete the self-reporting questionnaires
- understand the purpose of the study and having voluntarily submitted a consent form.

Before participation in the FTPs, we conducted several questionnaires to examine the general health status of the participants. We acquired basic demographic information that could affect cognitive and physical health and the Korean instrumental activities of daily living (K-IADL) to eliminate participants with functional disabilities owing to neurodegenerative diseases [46]. For the subgroup analysis, we used the KS-15, which is a short form of the Sasang constitution classification [44]. We also used a Korean version of the geriatric depression scale (GDS) [47,48] and a cold–heat pattern identification questionnaire; these will be discussed elsewhere.

Electrophysiological measurements were carried out at the baseline and after completing all the sessions of the FTPs. For the cognitive test, we used the Mini Mental-Status Examination for Dementia Screening (MMSE-DS) [49], an extensively used Korean version of the Mini Mental-Status Examination (MMSE) to assess the global cognitive status, and a neurocognitive test with the resting-state EEG. For the activation of the autonomic nervous system (ANS), we measured the HRV by photoplethysmography (PPG). Finally, we measured the bioimpedance to estimate the general body composition factors, including body fat mass; fat-free mass; total body water; and lower level information, such as resistance, reactance, and phase angle at 50 kHz. For comparison, the pre- and post-measurements were recorded for the CN as well.

Participants were recruited through advertisements and phone calls in cooperation with two county health centers (H-gu and S-gu) and a local senior citizen club (H senior club). Written informed consent was obtained from each subject prior to study participation. The study was approved by the Institutional Review Board of Chungbuk National University (IRB number: CBNU-201808-SB-678-01).

### 2.4. Electrophysiological Measurements

For the neurophysiological measurement, we used a wireless EEG device (neuroNicle FX2, LAXTHA, Inc., Korea) to measure the electrical activities in the prefrontal regions of Fp1 and Fp2 in the International 10/20 electrode system with a reference electrode on the right earlobe. The sampling rate was 250 Hz, the bandpass frequency was 3 to 43 Hz, and all contact impedances were kept below 10 kΩ. Subjects were seated in a comfortable position in a resting state with their eyes closed and muscles relaxed, and the EEG was recorded for five minutes in a quiet environment. A trained operator monitored the subject and EEG traces and alerted the subject whenever he/she showed signs of behavioral artifact or EEG drowsiness [50].

For the HRV measurement, we used a PPG device (ubpulse T1, LAXTHA, Inc., Korea). Subjects underwent PPG recordings on the fingertip for five minutes in a comfortable seated position in a resting state. The sampling rate was 250 Hz and the bandpass frequency was 0.3 to 10.6 Hz.

Bioelectrical impedance was measured using a bioimpedance analysis (BIA) device (InBody S-10, InBody, Korea), which measures impedance data with a direct segmental multifrequency method. We measured the impedance and reactance at three frequencies, namely, 5, 50, and 250 kHz. The phase angle (PhA) was defined by the angle between the impedance and reactance according to the following equation:

(1) $$\mathrm{PhA}=\arcsin(\mathrm{reactance}/|\mathrm{impendance}|)\times 180^{{}^{\circ}}/\pi$$

**Figure 1:**
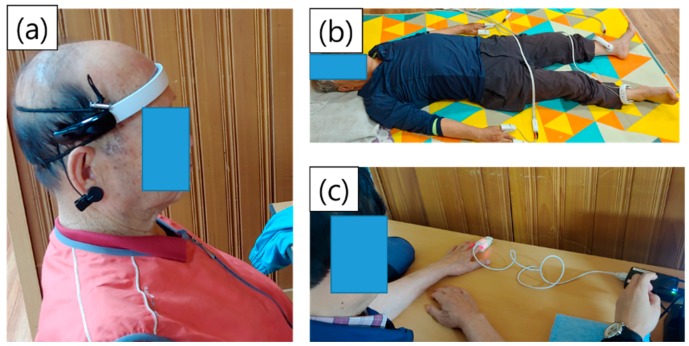
Photographs of the (**a**) electroencephalogram (EEG), (**b**) bioimpedance, and (**c**) photoplethysmography (PPG) measurement process.

Eight electrodes were used to measure five segmental impedance datapoints in the body; four were in contact with the thumbs and index fingers of each hand, and the other four were in contact with the interior and exterior sides of each ankle. The bioimpedance was measured with the subjects in a supine position. Four operators were adequately trained for the measurement of EEG, PPG, and bioimpedance. Figure 1 shows images of the EEG, bioimpedance, and HRV measurements, and Table 1 shows the analytical variables from each device.

### 2.5. Statistical Analysis

Statistical analyses were conducted using R statistical software (ver. 3.6.0) [51]. The significance level was set to α = 0.05 for all statistical tests (two-tailed). Before performing the analyses, two experts (J.C. and J.U.K.) screened the dataset for the resting-state EEG, bioimpedance, and HRV and ruled out inappropriate signals that should not be analyzed because of measurement error or pathophysiological factors such as arrhythmia. Missing values for the demographic characteristics and each biosignal were imputed using the multiple imputation (MI) method. MI was performed with the “mice” function provided by the “mice” package in R software, applying the option of the predictive mean matching algorithm [52].

The baseline characteristics of the participants according to each allocated FTP and the control group are summarized as the means and standard deviations (SDs) for continuous variables and the frequencies and proportions for categorical variables based on the available dataset (Table 2). The differences in baseline characteristics were investigated with one-way analysis of variance (ANOVA) or the chi-squared test for continuous and categorical variables, respectively.

The change in each biosignal outcome between the baseline (before attending the FTP) and the endpoint (after completing the FTP) was analyzed using the generalized linear model (GLM) with the identity function keeping the baseline values fixed. Several confounders, such as age, sex, education level, MMSE score, and daily activity hours, were also considered covariates in the GLM model (Table 3, Table 4 and Table 5). The mean change and its 95% confidence interval (CI) within each FTP and control group were provided according to each resting-state EEG, bioimpedance, or HRV variable. The multiple comparisons between two groups (change in BP vs. CN and change in WP vs. CN) were calculated to identify the mean difference in the change from the control group based on the t-statistics. *p*-values and 95% CIs related to the multiple tests were adjusted by Dunnett’s method. The effect sizes of the mean change in each FTP intervention and the mean difference in the change between the FTP and control groups provided in Table 3, Table 4, and Table 5 were calculated using the equation suggested by Rosnow et al. [53].

## 3. Results

**Figure 2:**
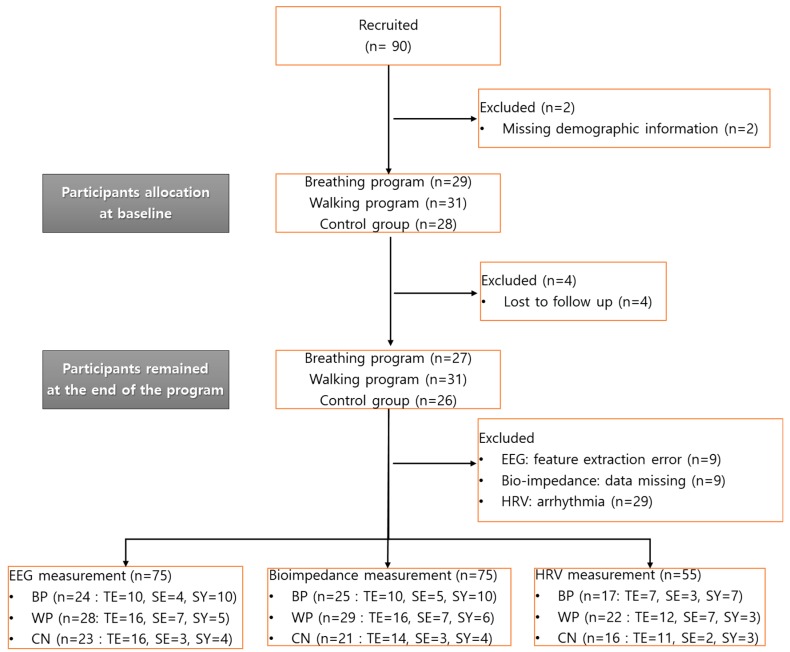
Flow chart of the study. HRV, heart rate variability; BP, guided-breathing meditation program; WP, walking program; CN, control; TE, Tae-Eum; SE, So-Eum; SY, So-Yang.

A total of 90 subjects were recruited for the study; 29 subjects were recruited from the S health center and allocated to the BP, 31 subjects were recruited from the H health center and allocated to the WP, and 30 subjects were recruited from the H senior club and allocated to the CN. Two subjects were excluded because of missing demographic information, and four subjects were lost to follow up during the FTPs. Finally, a total of 84 subjects remained for the biosignal measurements after finishing each FTP (27/31/26 subjects for the BP/WP/CN). Before analysis, two experts screened the dataset and eliminated inappropriate samples that could not be analyzed because of measurement errors or pathophysiological factors such as arrhythmia. As a result, the number of participants remaining for analysis in each dataset (EEG, bioimpedance, and HRV) was 74, 75, and 50, respectively, as shown in Figure 2; there was EEG data contamination for 10 participants, and there were missing bioimpedance measurements for 9 participants, while 34 participants were excluded from the HRV analysis because of arrhythmia.

### 3.1. Demographics

The participants’ demographics and baseline values are summarized in Table 2. There were significant differences in age, educational level, marital status, and MMSE scores among the BP, WP, and CN participants: in the order of BP, CN, and WP, the age decreased and the MMSE scores, education levels, and marital statuses increased. This tendency is in accordance with the general fact that the MMSE score is proportional to the duration of education and marital status and inversely proportional to age. There were no significant differences in other factors, such as body mass index (BMI), medical history, daily activity hours, sex, weight, height, or SC type.

### 3.2. Changes in Electrophysiology According to EEG, Bioimpedance, and HRV

**Figure 3:**
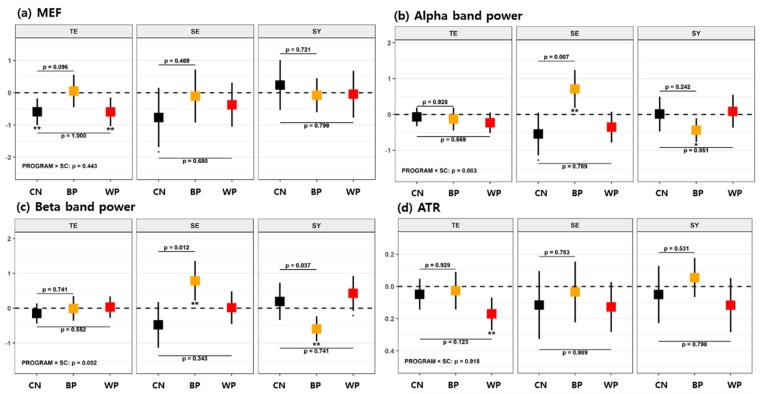
Mean changes in EEG variables after each of the forest therapy programs (FTPs) according to SC types for (**a**) median frequency (MEF), (**b**) Pα, (**c**) Pβ, and (**d**) alpha/theta ratio (ATR). Error bars represent 95% confidence intervals (CIs) for the mean changes. Asterisks below the lower confidence limits indicate the magnitude of statistical significance for the mean changes within each program group value (*p* < 0.1, * *p* < 0.05, ** *p* < 0.01, *** *p* < 0.001). The *p*-values noted in the figure are obtained from the multiple comparisons (BP vs. CN and WP vs. CN) for the group difference in the mean changes between the two groups. Dunnett’s method was used to adjust the type I error. Mean changes are adjusted for sex, age, education level, Mini Mental-Status Examination (MMSE), and daily activity hours.

**Figure 4:**
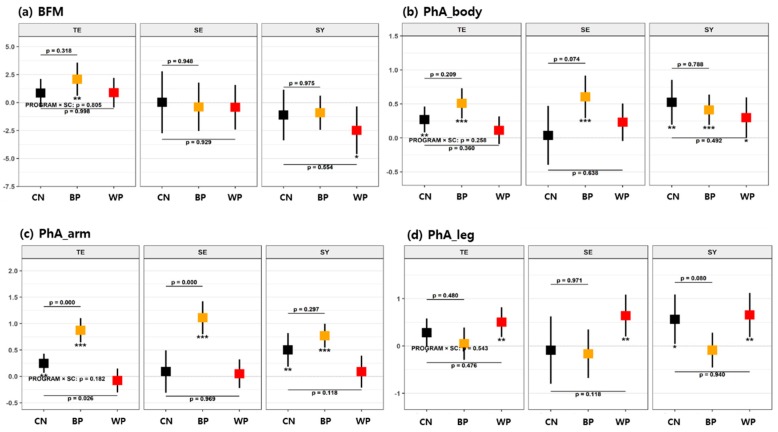
Mean changes in bioimpedance variables after each of the FTPs according to SC types for (**a**) body fat mass (BFM), (**b**) PhA_body, (**c**) PhA_arm, and (**d**) PhA_leg. The details are identical to those in Figure 3.

**Figure 5:**
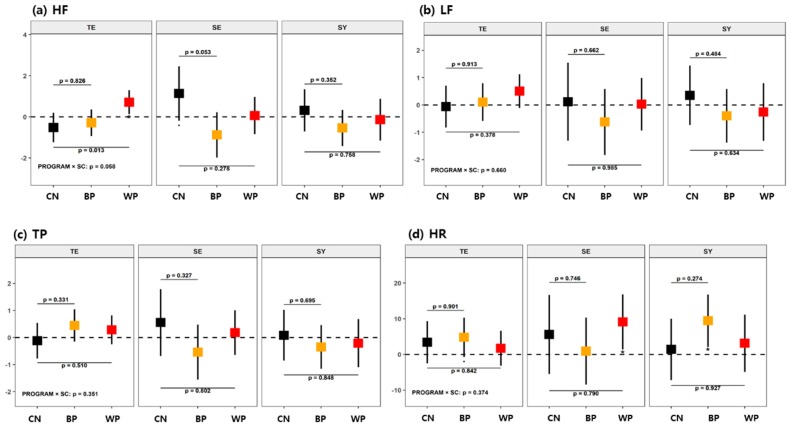
Mean changes in HRV variables after each of the FTPs according to SC type for (**a**) high frequency (HF), (**b**) low frequency (LF), (**c**) total power (TP), and (**d**) heart rate (HR). The details are identical to those in Figure 3.

We performed an analysis of covariance (ANCOVA) to examine the changes due to the forest therapy program interventions, changes after each FTP, and effective differences between each FTP and the CN. The analyzed variables in each dataset are described in Table 1. According to the detailed statistical methods described in Section 2.5, the changes in biomarkers due to each FTP were analyzed with a GLM, wherein several confounders, such as age, sex, education level, MMSE, and daily activity hours, were considered covariates; these models were generated to identify the mean difference in the change from the control group based on t-statistics. The test results are presented in Table 3, Table 4, and Table 5 for each FTP group analysis and in Figure 3, Figure 4, and Figure 5 for the subgroup analyses according to each SC type.

#### 3.2.1. Resting-State EEG

Table 3 shows the ANCOVA results of EEG variables of median frequency (MEF), the power of the alpha and beta bands (Pα & Pβ), and the ratio of Pα/Pβ (ATR). It shows, with respect to their baselines, a decrease in the MEF ($\bar{\delta }$ = −0.45 with 95% CI of (−0.78, −0.11), *p* < 0.01, and γ = 0.57) in the CN, no changes in the variables in the BP, and decreases in the MEF ($\bar{\delta }$ = −0.40 with 95% CI of (−0.77, −0.03), *p* < 0.05, and γ = 0.40) and ATR ($\bar{\delta }$ = −0.15 with 95% CI of (−0.23, −0.07), *p* < 0.001, and γ = 0.68) in the WP. In the multiple comparison analysis, no variable showed differences between groups in terms of *p*-values. In terms of the effect size, however, the BP showed a marginal increase in the MEF with Γ = 0.45 ($\bar{\Delta }=0.43$) compared with the CN, and the WP showed a marginal decrease in ATR with Γ = 0.44 ($\bar{\Delta }=-0.09$).

The effects of FTPs on cognition were analyzed in SC type subgroups, and the results are shown in Figure 3 (and details in Table A1, Appendix A). When only the TY-type participants were counted, the control group showed a decrease in MEF (*p* < 0.01 and γ = 0.74), and the WP group showed decreases in MEF (*p* < 0.01 and γ = 0.67) and ATR (*p* < 0.01 and γ = 0.84) with respect to their baselines. There were no mean differences in the variables between the WP and the CN, and there was a minor increase in MEF with the WP group compared with the CN group (*p* < 0.1 and Γ = 0.53). For the SE-type participants, the control group showed decreases in MEF (*p* < 0.1 and γ = 0.97) and Pα (*p* < 0.1 and γ = 1.06), but in contrast, the BP group showed increased Pα (*p* < 0.01 and γ = 1.35) and Pβ (*p* < 0.01 and γ = 1.38) with respect to their baselines. Moreover, mean increases in Pα (*p* = 0.07 and Γ = 0.80) and Pβ (*p* = 0.012 and Γ = 0.74) were found in the BP group compared with the CN group, but no significant changes in their mean values were observed between the WP and CN groups. For the SY-type participants, the BP group showed decreases in Pα (*p* < 0.05 and γ = 0.84) and Pβ (*p* < 0.01 and γ = 1.04), but the WP group showed increased Pβ (*p* < 0.1 and γ = 0.76). There was also a decrease in Pβ (*p* = 0.037 and Γ = 0.69) in the BP group compared with the CN group. Accordingly, the BP was effective at increasing the powers of the alpha and beta bands in the SE type, while no such effects were observed in the TE or SY type. On the other hand, the WP resulted in an improvement in beta band power for the SY type, but no such effect was found for the other SC types.

#### 3.2.2. Bioimpedance

Table 4 shows the ANCOVA results of bioimpedance variables such as the fat-free mass (FFM); body fat mass (BFM); % body fat (%BF); and impedances, reactances, and phase angles of the arms (Imp_arm, Reactance_arm & PhA_arm), legs (Imp_leg, Reactance_leg & PhA_leg), and whole body (PhA_body) (see list in Table 1). The overall changes in FFM, BFM, or %BF were not observed after any FTP or in the CN. However, there were increases in the whole body and segmental phase angles. Specifically, increases in PhA_body were observed in the BP group ($\bar{\delta }$ = 0.48 with 95% CI of (0.34, 0.63), *p* < 0.001, and γ = 1.30), the WP group ($\bar{\delta }$ = 0.20 with 95% CI of (0.03, 0.36), *p* < 0.05, and γ = 0.44), and the CN group ($\bar{\delta }$ = 0.29 with 95% CI of (0.14, 0.44), *p* < 0.001, and γ = 0.85), and a minor increase was found between the BP and the CN (Γ = 0.45), but there was no change between the WP and the CN groups.

More importantly, the segmental phase angles of the arms and legs showed opposite behaviors between different interventions. First, with respect to their baselines, a significant increase in the PhA_arm was observed in the BP group ($\bar{\delta }$ = 0.87 with 95% CI of (0.70, 1.03), *p* < 0.001, and γ = 2.13), and a significant increase in the PhA_leg was observed in the WP group ($\bar{\delta }$ = 0.60 with 95% CI of (0.35, 0.85), *p* < 0.001, and γ = 0.89), while relatively smaller increases in the PhA_arm and PhA_leg were observed in the CN group. Another interesting result was that no such increases were observed in the PhA_leg in the BP or the PhA_arm in the WP. Consequently, the PhA_arm increased significantly in the BP group compared with the CN group ($\bar{\Delta }=0.58$ with 95% CI of (0.33, 0.84), *p* < 0.001, and Γ = 1.29), while it decreased in the WP group compared with the CN group ($\bar{\Delta }=$−0.27 with 95% CI of (−0.51, −0.03), *p* < 0.05, and Γ = 0.64). In contrast, the PhA_leg increased significantly in the WP group compared with the CN group ($\bar{\Delta }=0.31$ with 95% CI of (−0.04, 0.67), *p* < 0.1, and Γ = 0.50), while it decreased in the BP group compared with the CN group ($\bar{\Delta }=$−0.35 with 95% CI of (−0.71, 0.23), *p* < 0.1, and Γ = 0.54).

The contrasting behaviors of segmental phase angles are mostly caused by the changes in reactance values rather than impedance values. For instance, small increases were found in the Imp_arm in the BP group ($\bar{\delta }/{\bar{X}}_{B}$ = 4.1%) and in the Imp_leg ($\bar{\delta }/{\bar{X}}_{B}$ = 4.7%) in the WP group with respect to their baselines; however, there were no significant differences from the CN group. Simultaneously, larger increases were found in the Reactance_arm ($\bar{\delta }/{\bar{X}}_{B}$ = 22.5%) in the BP group, in the Reactance_leg ($\bar{\delta }/{\bar{X}}_{B}$ = 15.4%) in the WP group, and increases in the Reactance_arm ($\bar{\delta }/{\bar{X}}_{B}$ = 5.5%) and Reactance_leg ($\bar{\delta }/{\bar{X}}_{B}$ = 7.9%) in the control group. Because the phase angle is the ratio between reactance and impedance (see Equation (1)), the increases in the segmental phase angles were mostly owing to the increases in the segmental reactance levels of the arm and leg.

The effects of FTPs on bioimpedance changes were analyzed by SC-type subgroup. In Figure 4 (see also Table A2, Appendix A), we only presented the variables that showed the SC-specific changes. Unlike the behaviors of the EEG variables, there were no contrasting behaviors between the different SC subgroups. BFM increased for the TE-type participants in the BP, while it decreased for the SY-type participants in the WP compared with their respective baselines. However, these values did not significantly differ from those of the CN participants. The phase angle of the whole body (PhA_body) increased irrespective of the SC type for all FTPs, showing no significant differences from the CN participants except for the SE-type participants in the BP (*p* < 0.1 and γ = 0.56).

More importantly, irrespective of the SC type, the within-group analysis showed that the PhA_arm increased in the BP group and the PhA_leg increased in the WP group compared with the baseline. Among the BP participants, significant increases in the PhA_arm were observed in the TE-type (*p* < 0.001 and Γ = 1.08) and SE-type participants (*p* < 0.001 and Γ = 1.09) in reference to the CN, while a decrease in the PhA_leg was observed in the SY type (*p* < 0.1 and Γ = 0.59). On the other hand, among the WP participants, there was no intergroup difference in the PhA_leg, but there was a decrease in the PhA_arm in the TE type (*p* < 0.05 and Γ = 0.66) compared with the CN.

#### 3.2.3. Heart Rate Variability

The changes in the HRV variables, such as the spectral powers of high frequency (HF), low frequency (LF), total power (TP = HF + LF + VLF), and heart rate (HR), were tested by ANCOVA and are shown in Table 5. The results showed a decrease in HF ($\bar{\delta }$ = −0.46 with 95% CI of (−0.98, 0.05), *p* < 0.1, and γ = 0.46) and an increase in HR ($\bar{\delta }$ = 5.97 with 95% CI of (1.82, 10.11), *p* < 0.005, and γ = 0.73) in the BP, but no changes in variables in the WP and the CN compared with their respective baseline values. Compared with the CN participants, the WP participants showed a minor increase in HF, with a moderate effect size (Γ = 0.48, but *p* > 0.1).

The effects of FTPs on the HRV changes were analyzed by SC subgroup, as shown in Figure 5 (see also Table A3, Appendix A). For the TE-type participants, the results showed increased HF in the WP compared with the CN (*p* = 0.013 and Γ = 1.02). For the SE-type participants, a minor decrease in HF was found in the BP compared with the CN (*p* = 0.053 and Γ = 0.79). No group differences were found in other variables. Intragroup differences were also found. For the SE-type participants, HR (*p* < 0.05 and γ = 1.07) increased with the WP. For the TE- and SY-type participants, HR (*p* < 0.1 and γ = 0.62 for TE, and *p* < 0.05 and γ = 1.17 for SY) increased with BP. Because an increase in HF indicates activated parasympathetic nerve activity [54], the WP was found to be effective in the TE-type participants. The increases in HR indicate upregulated blood flow; the upregulated effect was observed for the SE type in the WP and the SY type in the BP.

## 4. Discussion

The major aim of this study was to improve the neurocognitive abilities, autonomic nervous activities, and metabolic functions, and thus eventually prevent dementia, in older people via FTPs that could be carried out in nearby urban forests. In composing the FTPs, we assumed that distinct benefits could be induced by different characteristics of the program and that the benefits may vary with the psychophysiological traits of the participants. To test these assumptions, we first developed two FTPs with distinct characteristics. The BP was designed to induce deep breathing with postures that could induce the cervical spine to achieve optimal alignment to activate therapeutic Qi and blood flow through the meridian system and to increase cerebral blood flow, improving cognitive function while maintaining a low profile of other physical activities. The WP was designed to cause participants to walk actively to an extent that might induce sweating. Second, to test the personality-dependent benefits, we employed the concepts of SCM, in which individuals belonged to one of three different SC types with different psychological preferences and physiological susceptibilities to different types of physical activities.

In the forest therapy field, HRV is the most commonly used electrophysiological measurement to test ANS and cardiovascular function [12], and compared with walking in an urban area, walking in a forest generally has relaxing effects, with increased parasympathetic nervous system activity [16,55,56]. As advancing the measurement and signal processing techniques, portable EEG devices were recently implemented to assess neurocognitive activities stimulated by walking in the forest [57,58]. The bioimpedance analysis (BIA) technique was commercialized as a quick, portable, and reliable measurement of body composition, such as the FFM, fat mass, and total body water [59]. It is also a reliable estimator of cellular metabolism, such as intracellular water content, extracellular water content, and changes in intracellular pH and phosphocreatine [60]. This technique is not yet commonly implemented as a tool to evaluate the effect of forest therapy, as body composition does not change in a short period of time. However, in a repetitive FTP over a number of weeks, the body composition and cellular metabolic states are susceptible to change, and the BIA method can be a plausible tool to evaluate the therapeutic effects in the forest [23].

The implementation of 11 sessions over 11 weeks resulted in some beneficial effects with regard to neural activity in terms of EEG biomarkers; the ANS in terms of HRV biomarkers; and, for the first time, on bioimpedance in terms of the segmental phase angles. In terms of the EEG and HRV variables, compared with the CN, we observed a marginal increase in MEF among the EEG variables for the BP participants (effect size = 0.45, *p* > 0.1) and a marginal increase in HF among the HRV variables for the WP participants (effect size = 0.48, *p* > 0.1). A long history of literature supports that individuals with cognitive decline showed decreases in the MEF and ATR [50], and decreases in alpha wave activity and beta wave power are observed in the early stage of Alzheimer’s disease [61,62,63]. In terms of the effect size calculations based on Cohen’s d, the BP and WP showed possible beneficial effects for neural activity and for activation of the parasympathetic nervous response, respectively. Because the *p*-values are above the level of statistical significance, however, the results need to be confirmed by further study.

In terms of the bioimpedance variables, the PhA of the arms was increased largely for the BP participants (effect size = 1.29, *p* < 0.001), and the PhA of the legs was increased for the WP participants (effect size = 0.50, *p* < 0.1) compared with the CN. In contrast to the sensitive changes in total/segmental PhAs and related impedance and reactance values, no such changes were observed in FFM, BFM, or %BF. Recently, increases in phase angle by resistance training (RT) for older individuals have been reported by a series of works [64]. In particular, based on a progressive RT for 12 weeks (three times/week), Souza et al. reported increases in %BF, skeletal muscle mass, total body water, and PhA; among the observed variables, the PhA presented the highest relative effect size and statistical significance with respect to the differences between pre- and post-intervention and between training and control groups [65]. An emerging number of studies investigated the PhAs of BIA as a biomarker for various diseases and physiological conditions down to the cellular level. In particular, the PhAs at 50 kHz were reported to be lower in patients with diabetes mellitus [66], in patients with malnutrition [67], in patients at risk of death in the intensive care unit (ICU) [68], in people with lower FFM values, in patients with chronic obstructive pulmonary disease [69], and in cancer patients with lower quality of life and malnutrition [70]. These previous reports commonly indicate that changes in health conditions towards metabolic malnutritional states are related to reduced PhAs. Therefore, bioimpedance PhAs at 50 kHz may work as a prognostic biomarker of the FTP effects with respect to the recovery of cellular metabolism; the increased PhA in the arms for the BP participants may indicate increased metabolism in the upper body parts owing to the BP, and the increased PhA in the legs for the WP participants may indicate increased metabolism in the lower body parts owing to the WP. 

In the subgroup analysis by SC type, constitution-specific effects appeared in the EEG and HRV biomarkers. The SE-type participants gained benefits in neural activity (increased powers of alpha and beta waves) from the BP, and the SY-type participants obtained an increase in beta band power from the WP. With respect to the HRV biomarkers, the TE-type participants showed a relaxation effect (increased parasympathetic nerve activity) from the WP. Slightly upregulated blood flow effects were observed among the SE-type participants in the WP- and SY-type participants in the BP. According to a recent systematic review, the TE type has a low threshold for parasympathetic activation [42], which may explain why a moderate level of walking in the forest effectively induced elevated parasympathetic nerve activity in the TE type. However, the constitution-specific effects were not observed in the behavior of bioimpedance PhAs; for all TE-, SE-, and SY-type participants, the BP increased the average PhA of the arms, and the WP increased the average PhA of the legs.

Recent publications on SCM show that different SC types are characterized with different susceptibilities to metabolic syndrome. Compared with other SC types (especially with respect to the SE type), the TE type is characterized by an attenuated metabolic rate down to the cellular level [71] with suspected reduction in mitochondrial metabolism [72], and most likely as a consequence, the TE type is highly associated with an increased risk of general obesity and abdominal obesity [73], and individuals of the TE type have higher blood pressures, glucose metabolism values, and lipid profile levels, which impose a higher risk of type 2 diabetes [74]. The SC type itself was reported to be a risk factor for metabolic syndrome (TE > SY > SE) [75], and the SE type was a risk factor for irritable bowel syndrome [76]. FTPs can distinctively account for participants’ age, sex, and pathological and psychophysiological features. We showed that the SCM could be a candidate for such a tailored approach. To our knowledge, this is the first report of such a tailored approach in the field of forest therapy or exercise-based interventions for healthcare; we do not have systematic support from previous publications, and subsequent works should be followed to develop more practical FTPs accounting for participants’ psychophysiological traits.

There are some limitations of this study. Age, education level, and MMSE scores between the BP, WP, and CN participants were not well matched, which are known factors affecting neurocognitive status. More frequently used assessment tools to evaluate the effects of forest therapy with regard to the quality of life or profile of mood states could help evaluate more integrative effects. For the classification of the SC types, for the sake of convenience, we used only a short form (KS-15), the accuracy of which was reported to be approximately 63%; therefore, the subgroup analysis contains a moderate level of misclassification errors in the SC-type grouping [44]. In the subgroup analysis according to SC types, some cells had only a few samples, which might have resulted in biased findings. Taking into account that this work is only a feasibility study to check the possibility of different phenotypic responses to different therapeutic modalities in the forest, further studies should be designed to include more participants in each phenotype. There was no intervention for the control group, and designs for a future study should include an equivalent level of indoor exercise for the control group to investigate additional benefits due to performing the activities in the forest.

## 5. Conclusions

In conclusion, both the BP and the WP had health benefits for the elderly population in terms of neurophysiology, HRV, and bioimpedance, and the beneficial effects varied depending on the characteristics of the FTPs and on the SC types. In particular, the BP was effective at increasing the phase angle of the upper limbs, and the WP was effective at increasing the phase angle of the lower limbs. In the subgroup analysis according to the SCM typology, the BP was beneficial in increasing the powers of the alpha wave or beta wave in the SE type, and the WP was effective in increasing the beta wave power for the SY type. Moreover, the WP elevated the parasympathetic nervous system activity in the TE-type participants. These constitution-specific variations were not observed in the bioimpedance biomarkers. This result suggests that forest therapy can be effective at preventing dementia and that the beneficial effects can be maximized when the therapeutic program accounts for the participant’s psychological and physiological traits. To confirm the results of this feasibility study, more sessions with longer durations and a greater number of participants will be needed.

## Figures and Tables

**Table 1:** Datasets and characteristic variables to be analyzed. EEG, electroencephalogram; PPG, photoplethysmography.

Dataset	Variable	Explanation
EEG	MEF [Hz]	Median frequency: the median frequency in the dominant intrinsic oscillatory frequency band of 4–13 Hz of the power spectrum
	Pα [μV^2^]	Alpha band power: The spectral power integrated over the frequency range between 8 and 13 Hz (natural logarithmic scale)
	Pβ [μV^2^]	Beta band power: The spectral power integrated over the frequency range between 13 and 30 Hz (the natural logarithmic scale)
	ATR	Alpha/theta ratio: the power ratio of alpha rhythms (8–13 Hz) to theta rhythms (4–8 Hz)
Bioimpedance	FFM [kg]	Fat-free mass
	BFM [kg]	Body fat mass
	%BF [%]	Percent body fat (body fat/whole body mass)
	PhA_body	Phase angle of the whole body = (reactance of the whole body)/(impedance of the whole body)
	Imp_arm [Ω]	Impedance averaged over both arms
	Imp_leg [Ω]	Impedance averaged over both legs
	Reactance_arm [Ω]	Reactance averaged over both arms
	Reactance_leg [Ω]	Reactance averaged over both legs
	PhA_arm	Phase angle of both arms
	PhA_leg	Phase angle of both legs
HRV	HF [msec^2^]	Spectral power in the high frequency (HF) range of HRV (0.15–0.4 Hz)
	LF [msec^2^]	Spectral power in the low frequency (LF) range of HRV (0.04–0.15 Hz)
	%LF	LF power/(LF+HF power)
	HR [bpm]	Heart rate

**Table 2:** Demographic information. SC, Sasang constitutional; HRV, heart rate variability; TE, Tae-Eum; SE, So-Eum; SY, So-Yang; MMSE, Mini Mental-Status Examination; BMI, body mass index; FTP, forest therapy program.

Demographic Variable	Control Group	Breathing Program	Walking Program	*p*-Value
N (%)	28 (31.8%)	29 (33.0%)	31 (35.2%)	
Missing cases				
EEG	6 (20.7%)	5 (17.2%)	3 (9.7%)	0.486
HRV	16 (61.5%)	13 (44.8%)	9 (29.0%)	0.413
Bioimpedance	8 (27.6%)	4 (13.8%)	2 (6.5%)	0.075
SC Type				0.214
TE	18 (64.3%)	12 (41.4%)	16 (51.6%)	
SE	3 (10.7%)	5 (17.2%)	8 (25.8%)	
SY	7 (25.0%)	12 (41.4%)	7 (22.6%)	
Sex: Female	20 (69.0%)	22 (75.9%)	28 (90.3%)	0.118
Age [yr]	74.4 ± 4.9	78.5 ± 6.9	72.9 ± 6.2	0.002
Height [cm]	154.9 ± 6.6	153.3 ± 10.2	151.9 ± 5.5	0.325
Weight [kg]	60.8 ± 8.1	58.4 ± 9.7	56.5 ± 8.6	0.178
BMI [kg/m^2^]	25.3 ± 3.2	23.8 ± 2.9	24.4 ± 3.3	0.168
MMSE	25.3 ± 3.5	23.5 ± 4.0	26.3 ± 4.1	0.024
Smoking: Yes	1 (3.6%)	4 (13.8%)	1 (3.2%)	0.191
Alcohol: Yes	1 (3.6%)	3 (10.3%)	2 (6.5%)	0.595
Religion: Yes	22 (78.6%)	15 (51.7%)	20 (64.5%)	0.105
Marital status: Married	14 (50.0%)	4 (13.8%)	16 (51.6%)	0.004
Education level				0.005
None	3 (10.7%)	12 (41.4%)	1 (3.2%)	
1~3 years	2 (7.1%)	3 (10.3%)	7 (22.6%)	
4~6 years	13 (46.4%)	10 (34.5%)	10 (32.3%)	
7~9 years	3 (10.7%)	1 (3.4%)	2 (6.5%)	
More than 10 years	7 (25.0%)	3 (10.3%)	11 (35.5%)	
Medical history				
Hypertension	13 (46.4%)	17 (58.6%)	15 (48.4%)	0.609
Diabetes	5 (17.9%)	7 (24.1%)	5 (16.1%)	0.714
Dyslipidemia	13 (46.4%)	11 (37.9%)	8 (25.8%)	0.253
Arthritis	15 (53.6%)	17 (58.6%)	12 (38.7%)	0.275
Cerebrovascular disease	1 (3.6%)	2 (6.9%)	1 (3.2%)	0.758
Depression	1 (3.6%)	2 (6.9%)	0 (0.0%)	0.338
Parkinson’s disease	0 (0.0%)	0 (0.0%)	1 (3.2%)	0.395
Etc.	5 (17.9%)	13 (44.8%)	8 (25.8%)	0.071
Visits to forest [per month]	2.9 ± 5.1	1.4 ± 5.6	1.8 ± 3.7	0.491
Willing to participate in an FTP	24 (85.7%)	27 (93.1%)	31 (100.0%)	0.094
Daily activity hours [hour/day]	1.3 ± 1.7	0.9 ± 1.0	2.1 ± 3.2	0.114

Data are summarized as the means ± SDs for continuous variables and as the frequencies and proportions for categorical variables. *p*-values were derived from a one-way analysis of variance (ANOVA) test for continuous variables and a chi-squared test for categorical variables.

**Table 3:** Changes in EEG variables after the forest therapy programs (FTPs) (analysis of covariance (ANCOVA) results). BP, guided-breathing meditation program; WP, walking program; CI, confidence interval.

EEG	Control			Breathing Program			Walking Program			BP–CN		WP–CN	
Variable	${\bar{X}}_{B}$	$\bar{\delta }$ (95% CI)	γ	${\bar{X}}_{B}$	$\bar{\delta }$ (95% CI)	γ	${\bar{X}}_{B}$	$\bar{\delta }$ (95% CI)	γ	$\bar{\Delta }$ (95% CI)	Γ	$\bar{\Delta }$ (95% CI)	Γ
MEF [Hz]	8.47	−0.45 ** (−0.78, −0.11)	**0.57**	8.40	−0.02 (−0.38, 0.34)	0.02	8.40	−0.40 * (−0.77, −0.03)	**0.40**	0.43 (−0.11, 0.97)	**0.45**	0.05 (−0.48, 0.57)	0.05
$P\alpha \;\left[{\mu V}^{2}\right]$	2.71	−0.10 (−0.33, 0.13)	0.18	3.00	−0.17 (−0.42, 0.08)	0.28	2.65	−0.15 (−0.42, 0.11)	0.22	−0.07 (−0.45, 0.31)	0.11	−0.06 (−0.42, 0.31)	0.09
$P\beta \;\left[{\mu V}^{2}\right]$	1.86	−0.10 (−0.37, 0.16)	0.17	2.31	−0.16 (−0.44, 0.12)	0.24	2.09	0.13 (−0.16, 0.42)	0.17	−0.06 (−0.49, 0.37)	0.08	0.23 (−0.18, 0.64)	0.32
ATR	1.25	−0.06 (−0.13, 0.02)	0.33	1.18	0.00 (−0.08, 0.08)	0.02	1.22	−0.15 *** (−0.23, −0.07)	**0.68**	0.06 (−0.06, 0.18)	0.29	−0.09 (−0.21, 0.03)	**0.44**

The changes in variables after each FTP was analyzed using a generalized linear model (GLM), where ${\bar{X}}_{B}$ is the mean value at baseline and $\bar{\delta }$ (95% CI) and γ are the mean (95% confidence interval) and effect size, respectively, of the difference after each FTP or the CN in an equivalent time interval. Multiple comparisons between the two groups (change in BP vs. CN and WP vs. CN) were conducted to identify the mean difference in the change from the CN group based on t-statistics, where $\bar{\Delta }$ and Γ are the mean difference and effect size, respectively, of each FTP (BP or WP) relative to the CN. *p*-values (*p* < 0.1, * *p* < 0.05, ** *p* < 0.01, *** *p* < 0.001) and 95% CIs related to the multiple tests were adjusted by Dunnett’s method. Effect sizes were calculated by the Rosnow method.

**Table 4:** Changes in the bioimpedance variables after the FTPs (analysis of covariance (ANCOVA) results). The details are identical to those in Table 3. FFM, fat-free mass; BFM, body fat mass; %BF, % body fat.

Bioimpedance	Control			Breathing Program			Walking Program			BP–CN		WP–CN	
Variable	${\bar{X}}_{B}$	δ (95% CI)	γ	${\bar{X}}_{B}$	$\bar{\delta }$ (95% CI)	γ	${\bar{X}}_{B}$	$\bar{\delta }$ (95% CI)	γ	$\bar{\Delta }$ (95% CI)	Γ	$\bar{\Delta }$ (95% CI)	Γ
FFM [kg]	40.70	0.72 (−0.30, 1.74)	0.31	37.47	−0.26 (−1.26, 0.74)	0.10	38.28	0.20 (−0.92, 1.31)	0.07	−0.98 (−2.52, 0.55)	0.36	−0.53 (−2.01, 0.96)	0.20
BFM [kg]	20.48	0.26 (−0.79, 1.30)	0.11	20.21	0.62 (−0.43, 1.67)	0.24	18.72	0.02 (−1.15, 1.18)	0.01	0.36 (−1.30, 2.02)	0.12	−0.24 (−1.88, 1.40)	0.08
%BF [%]	33.00	−0.36 (−1.85, 1.13)	0.11	34.48	0.75 (−0.76, 2.26)	0.20	32.25	−0.41 (−2.12, 1.31)	0.09	1.11 (−1.26, 3.47)	0.26	−0.04 (−2.39, 2.30)	0.01
PhA_body	5.38	0.29 *** (0.14, 0.44)	**0.85**	4.91	0.48 *** (0.34, 0.63)	**1.30**	5.42	0.20 * (0.03, 0.36)	**0.44**	0.19 (−0.05, 0.43)	**0.45**	−0.09 (−0.32, 0.13)	0.24
Imp_arm [Ω]	343.69	3.22 (−4.38, 10.82)	0.18	346.11	14.25 *** (6.54, 21.96)	**0.74**	347.20	−1.19 (−9.63, 7.25)	0.05	11.03 (−1.01, 23.08)	**0.51**	−4.41 (−16.13, 7.32)	0.21
Imp_leg [Ω]	168.44	3.41 (−2.91, 9.74)	0.24	180.63	−3.86 (−10.13, 2.40)	0.25	168.65	8.76 * (1.65, 15.87)	**0.46**	−7.28 (−17.27, 2.71)	**0.41**	5.35 (−4.37, 15.07)	0.31
Reactance_arm [Ω]	31.45	1.74 * (0.29, 3.19)	**0.52**	28.07	6.32 *** (4.70, 7.95)	**1.55**	33.92	−0.56 (−2.18, 1.06)	0.13	4.58 *** (2.14, 7.03)	**1.05**	−2.30 * (−4.57, −0.02)	**0.57**
Reactance_leg [Ω]	15.52	1.23 * (0.23, 2.23)	**0.53**	16.24	−0.37 (−1.39, 0.65)	0.14	14.58	2.25 *** (1.12, 3.39)	**0.74**	**−1.60 (−3.21, 0.01)**	**0.56**	1.02 (−0.56, 2.60)	0.37
PhA_arm	5.28	0.28 *** (0.14, 0.43)	**0.85**	4.66	0.87 *** (0.70, 1.03)	**2.13**	5.63	0.01 (−0.17, 0.20)	0.03	0.58 *** (0.33, 0.84)	**1.29**	−0.27 * (−0.51, −0.03)	**0.64**
PhA_leg	5.35	0.29 * (0.06, 0.52)	**0.54**	5.16	−0.06 (−0.30, 0.17)	0.11	4.97	0.60 *** (0.35, 0.85)	**0.89**	**−0.35 (−0.71, 0.02)**	**0.54**	**0.31 (−0.04, 0.67)**	**0.50**

**Table 5:** Changes in HRV variables after FTPs (ANCOVA results). The details are identical to those in Table 3. HF, high frequency; LF, low frequency; TP, total power; HR, heart rate.

HRV	Control			Breathing Program			Walking Program			BP–CN		WP–CN	
Variable	${\bar{X}}_{B}$	$\bar{\delta }$ (95% CI)	γ	${\bar{X}}_{B}$	$\bar{\delta }$ (95% CI)	γ	${\bar{X}}_{B}$	$\bar{\delta }$ (95% CI)	γ	$\bar{\Delta }$ (95% CI)	Γ	$\bar{\Delta }$ (95% CI)	Γ
HF [msec^2^]	3.88	−0.13 (−0.69, 0.44)	0.13	4.27	**−0.46 (−0.98, 0.05)**	**0.46**	4.61	0.41 (−0.09, 0.92)	0.35	−0.34 (−1.22, 0.55)	0.28	0.54 (−0.31, 1.39)	**0.48**
LF [msec^2^]	3.50	0.00 (−0.55, 0.56)	0.00	4.03	−0.16 (−0.68, 0.35)	0.16	3.99	0.28 (−0.22, 0.78)	0.24	−0.17 (−1.05, 0.72)	0.14	0.27 (−0.56, 1.11)	0.25
%LF	46.89	−1.38 (−6.31, 3.55)	0.16	48.25	1.48 (−3.19, 6.14)	0.16	46.50	−1.38 (−5.90, 3.14)	0.13	2.86 (−5.00, 10.71)	0.26	0.00 (−7.40, 7.40)	0.00
TP [msec^2^]	5.47	−0.04 (−0.53, 0.46)	0.04	5.88	0.05 (−0.40, 0.50)	0.06	6.14	0.17 (−0.27, 0.62)	0.17	0.09 (−0.69, 0.86)	0.08	0.21 (−0.54, 0.96)	0.21
HR [bpm]	68.93	2.87 (−1.58, 7.33)	0.38	67.64	5.97 ** (1.82, 10.11)	**0.73**	64.17	3.02 (−1.04, 7.09)	0.32	3.09 (−3.85, 10.04)	0.32	0.15 (−6.61, 6.91)	0.02

## Appendix A

Owing to the space limitation and to provide results with reduced visual complexity, we presented the constitution-specific results only with figures that did not contain the essential information with the effect size. Here, the information supplementary to Figure 3 through Figure 5 is presented in Table A1 through Table A3.

**Table A1:** Changes in EEG variables after FTPs according to SC types (ANCOVA results).

EEG Variable		Control			BP			WP			BP–CN		WP–CN	
	SC Type	${\bar{X}}_{B}$	$\bar{\delta }$ (95% CI)	γ	${\bar{X}}_{B}$	$\bar{\delta }$ (95% CI)	γ	${\bar{X}}_{B}$	$\bar{\delta }$ (95% CI)	γ	$\bar{\Delta }$ (95% CI)	Γ	$\bar{\Delta }$ (95% CI)	Γ
MEF [Hz]	TE	8.49	−0.59 ** (−1.01, −0.18)	0.74	8.62	0.05 (−0.46, 0.56)	0.06	8.59	−0.60 ** (−1.04, −0.15)	0.67	0.65 (−0.10, 1.39)	0.53	−0.00 (−0.65, 0.64)	0.00
	SE	8.36	−0.77 (−1.69, 0.15)	0.97	8.61	−0.10 (−0.93, 0.72)	0.13	8.06	−0.37 (−1.06, 0.31)	0.41	0.67 (−0.76, 2.09)	0.28	0.40 (−0.84, 1.63)	0.21
	SY	8.48	0.24 (−0.53, 1.01)	0.31	8.08	−0.08 (−0.61, 0.45)	0.10	8.30	−0.05 (−0.77, 0.68)	0.06	−0.32 (−1.40, 0.76)	0.19	−0.29 (−1.48, 0.91)	0.14
$P\alpha \;\left[{\mu V}^{2}\right]$	TE	2.85	−0.07 (−0.33, 0.19)	0.13	2.94	−0.13 (−0.45, 0.19)	0.25	2.62	−0.23 (−0.52, 0.05)	0.41	−0.06 (−0.53, 0.41)	0.08	−0.16 (−0.57, 0.25)	0.24
	SE	2.10	−0.54 (−1.14, 0.05)	1.06	3.36	0.71 ** (0.18, 1.24)	1.35	2.71	−0.35 (−0.78, 0.07)	0.63	1.26 ** (0.32, 2.20)	0.80	0.19 (−0.59, 0.97)	0.16
	SY	2.62	0.01 (−0.47, 0.50)	0.02	2.91	−0.44 * (−0.77, −0.11)	0.84	2.64	0.09 (−0.37, 0.54)	0.17	−0.45 (−1.13, 0.23)	0.43	0.08 (−0.68, 0.83)	0.06
$P\beta \;\left[{\mu V}^{2}\right]$	TE	1.96	−0.15 (−0.44, 0.14)	0.27	2.38	−0.01 (−0.36, 0.35)	0.01	2.02	0.03 (−0.28, 0.34)	0.05	0.15 (−0.38, 0.67)	0.17	0.18 (−0.26, 0.63)	0.24
	SE	1.15	−0.48 (−1.14, 0.18)	0.84	2.49	0.79 ** (0.22, 1.36)	1.38	2.22	0.01 (−0.45, 0.48)	0.02	1.26 * (0.25, 2.28)	0.74	0.49 (−0.38, 1.37)	0.37
	SY	2.01	0.19 (−0.34, 0.73)	0.36	2.16	−0.59 ** (−0.96, −0.23)	1.04	2.12	0.43 (−0.08, 0.93)	0.76	−0.79 * (−1.53, −0.04)	0.69	0.23 (−0.60, 1.06)	0.17
ATR	TE	1.28	−0.05 (−0.14, 0.05)	0.26	1.20	−0.03 (−0.14, 0.09)	0.15	1.25	−0.17 ** (−0.27, −0.07)	0.84	0.02 (−0.15, 0.19)	0.08	−0.12 (−0.27, 0.03)	0.49
	SE	1.24	−0.12 (−0.33, 0.10)	0.63	1.17	−0.03 (−0.22, 0.16)	0.18	1.18	−0.13 (−0.28, 0.03)	0.62	0.08 (−0.25, 0.41)	0.15	−0.01 (−0.29, 0.27)	0.03
	SY	1.14	−0.05 (−0.23, 0.13)	0.28	1.17	0.05 (−0.07, 0.18)	0.29	1.14	−0.12 (−0.28, 0.05)	0.62	0.11 (−0.14, 0.35)	0.28	−0.07 (−0.34, 0.21)	0.14

The changes in variables after each FTP were analyzed using a GLM, where ${\bar{X}}_{B}$ is the mean value at baseline and $\bar{\delta }$ (95% CI) and γ are the mean (95% confidence interval) and effect size, respectively, of the difference after each FTP or the CN in an equivalent time interval. Multiple comparisons between the two groups (change in BP vs. CN and WP vs. CN) were conducted to identify the mean difference in the change from the CN group based on t-statistics, where $\bar{\Delta }$ and Γ are the mean difference and effect size, respectively, of each FTP (BP or WP) relative to the CN. *p*-values (*p* < 0.1, * *p* < 0.05, ** *p* < 0.01, *** *p* < 0.001) and 95% CIs related to the multiple tests were adjusted by Dunnett’s method. Effect sizes were calculated by the Rosnow method.

**Table A2:** Changes in bioimpedance variables after FTPs according to SC types (ANCOVA results). The details are identical to those in Table A1.

Bioimpedance		Control			BP			WP			BP−CN		WP−CN	
Variable	SC Type	${\bar{X}}_{B}$	$\bar{\delta }$ (95% CI)	γ	${\bar{X}}_{B}$	$\bar{\delta }$ (95% CI)	γ	${\bar{X}}_{B}$	$\bar{\delta }$ (95% CI)	γ	$\bar{\Delta }$ (95% CI)	Γ	$\bar{\Delta }$ (95% CI)	Γ
BFM	TE	23.53	0.86 (−0.39, 2.10)	0.37	25.31	2.09 ** (0.61, 3.56)	0.90	22.67	0.89 (−0.44, 2.21)	0.33	1.23 (−0.87, 3.33)	0.35	0.03 (−1.90, 1.95)	0.01
	SE	14.26	0.03 (−2.74, 2.79)	0.01	16.65	−0.39 (−2.55, 1.78)	0.16	12.88	−0.42 (−2.42, 1.57)	0.16	−0.41 (−4.32, 3.49)	0.06	−0.45 (−4.00, 3.10)	0.08
	SY	14.47	−1.11 (−3.37, 1.15)	0.49	16.89	−0.91 (−2.43, 0.62)	0.38	15.02	−2.48 * (−4.61, −0.35)	0.95	0.20 (−2.80, 3.21)	0.04	−1.37 (−4.73, 1.99)	0.24
PhA_body	TE	5.55	0.27 ** (0.08, 0.46)	0.76	4.78	0.51 *** (0.29, 0.73)	1.45	5.53	0.11 (−0.09, 0.32)	0.28	0.24 (−0.10, 0.58)	0.42	−0.16 (−0.45, 0.13)	0.32
	SE	4.63	0.04 (−0.40, 0.47)	0.10	5.04	0.60 *** (0.29, 0.92)	1.73	5.13	0.23 (−0.05, 0.51)	0.63	0.57 (−0.05, 1.18)	0.56	0.19 (−0.36, 0.74)	0.22
	SY	5.33	0.52 ** (0.19, 0.85)	1.59	4.98	0.41 *** (0.19, 0.64)	1.17	5.47	0.30 * (0.00, 0.59)	0.82	−0.11 (−0.56, 0.34)	0.16	−0.23 (−0.73, 0.28)	0.27
PhA_arm	TE	5.40	0.25 ** (0.07, 0.43)	0.73	4.58	0.87 *** (0.64, 1.10)	2.41	5.75	−0.08 (−0.30, 0.15)	0.17	0.63 *** (0.28, 0.97)	1.08	−0.32 * (−0.62, −0.03)	0.66
	SE	4.83	0.09 (−0.31, 0.49)	0.26	4.68	1.11 *** (0.80, 1.42)	3.19	5.36	0.05 (−0.22, 0.32)	0.14	1.02 *** (0.45, 1.59)	1.09	−0.04 (−0.58, 0.49)	0.05
	SY	5.24	0.50 ** (0.18, 0.82)	1.57	4.73	0.77 *** (0.55, 0.99)	2.17	5.63	0.09 (−0.21, 0.39)	0.24	0.27 (−0.17, 0.71)	0.39	−0.41 (−0.91, 0.09)	0.50
PhA_leg	TE	5.68	0.28 (−0.02, 0.58)	0.50	4.98	0.05 (−0.29, 0.39)	0.09	5.06	0.50 ** (0.19, 0.82)	0.80	−0.23 (−0.74, 0.27)	0.27	0.22 (−0.26, 0.70)	0.27
	SE	3.89	−0.09 (−0.80, 0.62)	0.14	5.45	−0.16 (−0.68, 0.35)	0.29	4.86	0.64 ** (0.20, 1.08)	1.10	−0.08 (−1.13, 0.97)	0.04	0.73 (−0.15, 1.61)	0.53
	SY	5.32	0.56 * (0.04, 1.09)	1.08	5.20	−0.09 (−0.45, 0.28)	0.15	4.87	0.66 ** (0.19, 1.12)	1.15	−0.65 (−1.37, 0.06)	0.59	0.09 (−0.71, 0.89)	0.07

**Table A3:** Changes in HRV variables after FTPs according to SC types (ANCOVA results). The details are identical to those in Table A1.

HRV Variable		Control			BP			WP			BP–CN		WP–CN	
	SC Type	${\bar{X}}_{B}$	$\bar{\delta }$ (95% CI)	γ	${\bar{X}}_{B}$	$\bar{\delta }$ (95% CI)	γ	${\bar{X}}_{B}$	$\bar{\delta }$ (95% CI)	γ	$\bar{\Delta }$ (95% CI)	Γ	$\bar{\Delta }$ (95% CI)	Γ
HF [ms^2^]	TE	3.78	−0.52 (−1.23, 0.20)	0.56	4.18	−0.29 (−0.93, 0.36)	0.32	4.23	0.71 * (0.13, 1.30)	0.69	0.23 (−0.85, 1.32)	0.17	1.23 * (0.24, 2.22)	1.02
	SE	3.93	1.14 (−0.19, 2.46)	1.23	4.13	−0.88 (−1.98, 0.23)	0.93	4.80	0.06 (−0.84, 0.97)	0.06	−2.01 (−4.05, 0.03)	0.79	−1.07 (−2.81, 0.66)	0.54
	SY	4.09	0.31 (−0.71, 1.34)	0.36	4.51	−0.54 (−1.42, 0.34)	0.56	5.58	−0.13 (−1.15, 0.88)	0.13	−0.85 (−2.40, 0.69)	0.45	−0.45 (−2.14, 1.25)	0.21
LF [ms^2^]	TE	3.21	−0.06 (−0.83, 0.71)	0.06	3.58	0.10 (−0.59, 0.79)	0.11	3.76	0.51 (−0.11, 1.12)	0.46	0.16 (−0.99, 1.32)	0.11	0.56 (−0.50, 1.63)	0.44
	SE	3.48	0.12 (−1.31, 1.54)	0.12	4.53	−0.62 (−1.83, 0.58)	0.61	3.90	0.03 (−0.94, 0.99)	0.02	−0.74 (−2.99, 1.51)	0.26	−0.09 (−1.93, 1.75)	0.04
	SY	4.22	0.35 (−0.74, 1.44)	0.38	4.45	−0.40 (−1.38, 0.58)	0.37	4.86	−0.26 (−1.31, 0.80)	0.25	−0.75 (−2.41, 0.91)	0.37	−0.61 (−2.35, 1.14)	0.28
TP [ms^2^]	TE	5.35	−0.12 (−0.77, 0.54)	0.14	5.61	0.45 (−0.15, 1.04)	0.54	6.02	0.28 (−0.25, 0.82)	0.30	0.56 (−0.42, 1.55)	0.45	0.40 (−0.52, 1.33)	0.36
	SE	5.11	0.55 (−0.68, 1.79)	0.65	6.14	−0.54 (−1.55, 0.48)	0.62	6.04	0.18 (−0.64, 1.01)	0.20	−1.09 (−3.00, 0.81)	0.46	−0.37 (−1.97, 1.23)	0.20
	SY	5.97	0.09 (−0.85, 1.02)	0.11	6.16	−0.35 (−1.15, 0.46)	0.39	6.69	−0.21 (−1.10, 0.68)	0.24	−0.43 (−1.84, 0.98)	0.25	−0.30 (−1.79, 1.20)	0.16
HR [bpm]	TE	68.63	3.42 (−2.49, 9.32)	0.44	68.02	4.79 (−0.71, 10.29)	0.62	64.79	1.73 (−3.17, 6.63)	0.20	1.37 (−7.67, 10.41)	0.12	−1.69 (−10.04, 6.66)	0.17
	SE	70.54	5.59 (−5.48, 16.67)	0.72	70.60	0.93 (−8.46, 10.33)	0.12	63.63	9.11 * (1.40, 16.82)	1.07	−4.66 (−21.76, 12.44)	0.22	3.51 (−11.07, 18.10)	0.21
	SY	68.57	1.41 (−7.21, 10.02)	0.19	65.27	9.45 * (2.13, 16.77)	1.17	62.81	3.14 (−4.87, 11.15)	0.40	8.04 (−4.87, 20.95)	0.50	1.73 (−11.99, 15.46)	0.10
